# The use of biological membranes for correction of congenital malformations

**DOI:** 10.1007/s10561-022-10003-x

**Published:** 2022-04-06

**Authors:** C Marchetto, A Sgrò, P Gamba, D Trojan, C Pagliara, P Midrio

**Affiliations:** 1 Pediatric Surgery, Cà Foncello Hospital, Treviso, Italy; 2 Present Address: Pediatric Surgery, University of Padua, Padua, Italy; 3 Fondazione Banca dei Tessuti di Treviso - Onlus, Treviso, Italy

**Keywords:** Biological membranes, Biomaterials, Congenital malformations, Tissue Banks

## Abstract

Many congenital malformations often require a multidisciplinary and multistep surgical treatment, including the use of biological membranes. Aims of the study were to describe the use of these membranes for the correction of malformations, their clinical performance at follow-up, and patient's tolerance to them. The study included patients treated between 2009 and November 2020 in two referral centers. They were affected by abdominal wall defects (AWD), esophageal atresia/tracheo-esophageal fistula (EA/TEF), diaphragmatic hernia (CDH), spinal defects (SD), and anorectal malformations (ARM). The human origin membranes used during surgery were amniotic membrane, *fascia lata*, and pericardium provided by the local tissue bank and the porcine-derived membrane available on the market. Thirty-one patients were retrieved. The sample included 10 AWD, 7 EA/TEF, 5 CDH, 4 SD, 2 ARM, and 3 miscellaneous defects. The median age at repair was 139 days (range: 10,5–1494). The median follow-up was 1021 days (range: 485,5–1535). Two patients were lost at follow-up. The defects were successfully repaired and the membranes perfectly tolerated in 28/29 cases. In 1 case of CDH the *fascia lata* was replaced with a Goretex patch due to recurrence of the defect. This is the largest series on the use of biological membranes in congenital malformations. The variety of tissues allows to choose the best material for each malformation. The excellent tolerance and performance of this first series of patients encourage the use of these membranes to correct different type of malformations at any age.

## Introduction

Congenital malformations represent single or multiple defects of the morphogenesis of organs or entire body district. The prevalence at birth is 2–3% (1:50) and they often require a multidisciplinary approach (Gamba and Midrio 2014). When major congenital anomalies occur, surgical correction is mandatory. The primary endpoint is always the restoration of a normal anatomy; nevertheless, great attention needs to be paid to the functional and aesthetic aspects (Gamba and Midrio 2014). To achieve these targets, the use of different biological tissues may be required at surgery. This is a well-known and diffuse practice in many adult’s type of surgery, such as in case of post-traumatic reconstructive surgery or after tumor resections (Goto et al. 2020; Rifaat and Abdel Gawat 2005). However, in pediatrics the experience is more recent and limited, including mainly the treatment of burn injuries or in pediatric ophthalmology (Vloemans et al. 2014; Jirsova and Jones 2017).

The aims of this work, in a double-center setting, are to describe the use of biological membranes in the most frequent major congenital malformations and to evaluate both tissue’s performance and host’s tolerance to the biological graft at medium-term follow-up.

## Materials and methods

### Patients

A retrospective double-center cohort study was conducted on pediatric patients (< 18 years-old) treated for congenital anomalies between January 2009 and November 2020 who required the use of biological membranes. The study included patients who underwent surgery at the Pediatric Surgery Unit of Treviso and Padua affected by Abdominal Wall Defects (AWD), Esophageal Atresia (EA)/Tracheo-Esophageal Fistula (TEF), Congenital Diaphragmatic Hernia (CDH), Anorectal Malformations (ARM), Spinal Defects, and other conditions. Only patients with a follow-up of at least four months were considered.

Patients were retrieved using the ICD-9 coding system, clinical databases of both centers, and the registry of Fondazione Banca dei Tessuti di Treviso (FBTV). FBTV is a non-for-profit multitissues bank tasked with retrieval, processing, preserving, validating, and distributing the human tissues for clinical use. The suitability and safety of tissues are certified in accordance with the European and Italian Legislation.

Collected data were patients’ demographics, prenatal diagnosis, comorbidities, perioperative parameters (age and weight at the time of surgery), type of surgery, short and medium-term postoperative complications, and need for additional surgery.

Written consent was collected from parents for the use of biological membranes and analysis of data.

### Human tissues and porcine-derived membrane

The membranes are employed to recreate the anatomic barrier between structures originally separated (i.e. in case of a fistula) or to strengthen the interfaces between different organs when a high mechanical resistance is needed. The choice of a specific type of membrane is made by the surgeon upon the expected aim for its use. In this study four types of biological membranes were used: human amniotic membrane, human *fascia lata*, porcine-derived biological membrane, and human pericardium.

Pericardium and fascia are retrieved from cadaver donors while amniotic membrane from living donors. Donors were selected on the basis of strict criteria including directives for harvesting, processing and distributing tissues for transplantation as approved by the National Transplant Centre. The blood of the donors was also screened for HIV-1 and -2 antibodies, HTLV-1 and -2 antibodies, Hepatitis B Surface Antigen and Hepatitis B Core Antibody, Hepatitis C virus (HCV) and syphilis. Screening also included IgM/IgG antibodies against toxoplasmosis (only for amniotic membrane) and cytomegalovirus and nucleic acid amplification tests (NAT) for HIV, HBV and HCV. Several microbiological tests are performed to assess the suitability of the tissues. The antibiotic cocktail used to decontaminate human tissues distributed by FBTV is validated to avoid any risk of contamination (Montagner et al. 2018). Amniotic membrane, fascia lata and pericardium, after processing and decontamination protocol, are cryopreserved.

### Amniotic membrane

**Fig. 1:**
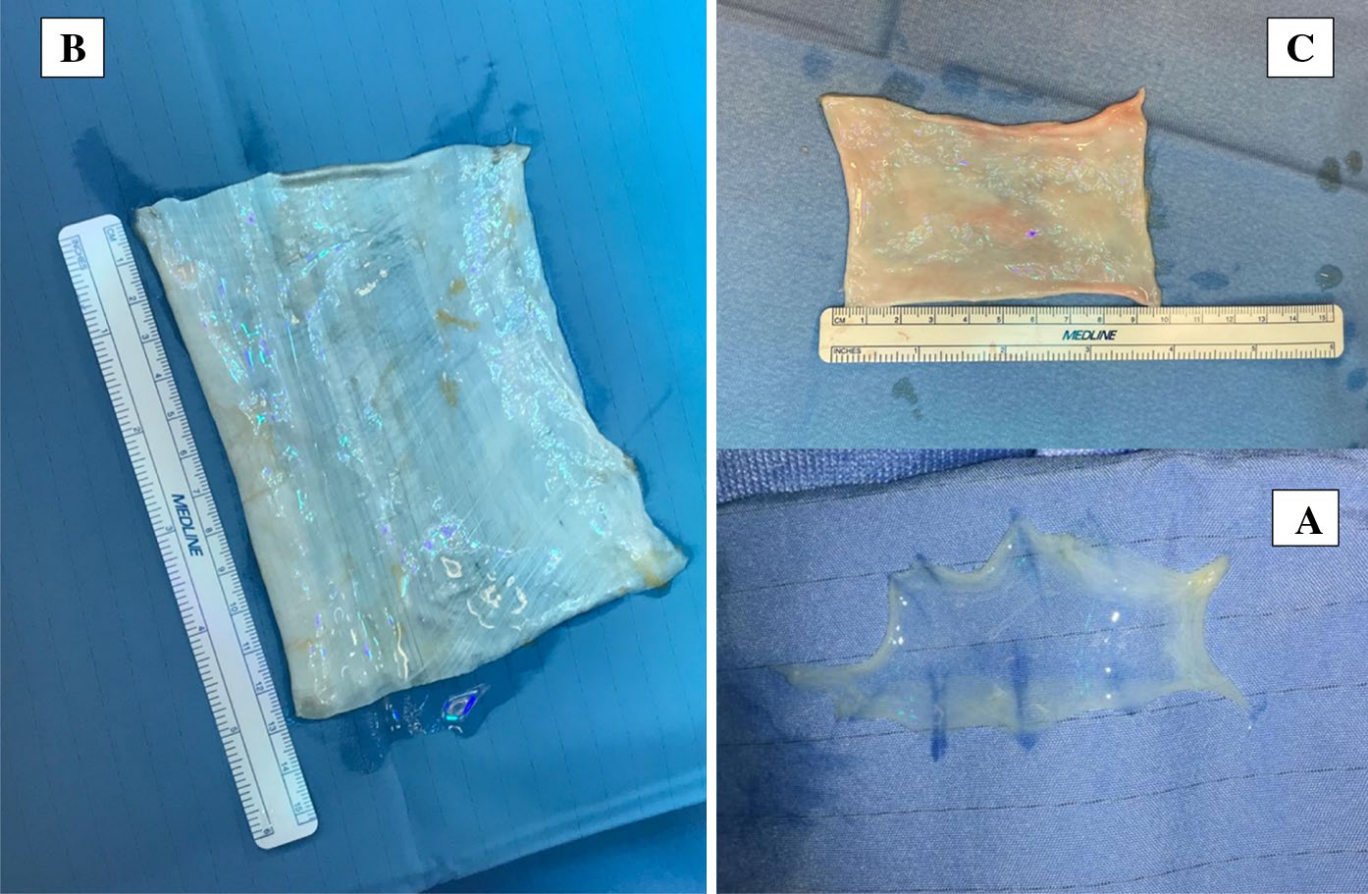
(A) Amniotic membrane. (B) Patch of *fascia lata.* (C) Pericardium

The amniotic membrane is extracted from donated placenta. (Fig. 1.A) Macroscopically, it appears as a thin transparent and elastic film that can be easily handled and sutured. It may act as a biological bandage, covering exposed and/or inflamed area, stimulating healing and reducing pain and discomfort, or as a substrate for adhesion and growth of epithelial cells, through secretion of various growth factors (Koizumi et al. 2000; Dua et al. 2004; Kurpakus et al. 1999). Some authors assumed that amniotic membrane may have anti-inflammatory properties releasing cytokines and stimulating the apoptosis of inflammatory cells (Dua et al. 2004; Espana et al. 2002; Tseng et al. 2004). Finally, it has been proved to reduce scarring and to protect the skin from infection (Dua et al. 2004; Kjaergaard et al. 2001).

### Fascia lata

The fascia lata is one of the strongest fascial layers, easy to obtain (Sugiyama et al. 2011) and used in many surgical fields. It mainly consists of fibrous components, after removal of muscular and adipose tissues during the processing, and its strength is unlikely to change (Suzuki et al. 2002) (Fig. 1.B).

### Pericardium

The pericardium consists of a serous and fibrous layer. This membrane is a resistant tissue applied in neurosurgery, plastic surgery, and general surgery (Fig. 1C).

### Porcine-derived biological membrane

The porcine membranes are absorbable biomaterials derived from porcine xenogenic extracellular matrix (ECM). They are mostly used in large abdominal wall defects (Zhang et al. 2011).

## Results

### Population

A total of 31 patients were included in the study, 17 females and 14 males, from 0 to 16 years-old. The median age at surgery was 139 days (interquartile range: 10,5–1494 days). Ten (32.2%) patients were affected by AWD, 7 (22,6%) by EA/TEF, 5 (16.1%) by CDH, and 2 (6.4%) by ARM cloaca type. SD were detected in 4 patients (12,9%): 3 myelomeningocele and 1 Currarino syndrome. Finally, we included an early-onset inflammatory bowel disease with IL-10 receptor deficit, a bronchogenic cyst, and an extradural hematoma related to rupture of a congenital artero-venous malformation. The biological membranes employed were: 14 amniotic membranes (45%), 11 fascia lata (35%), 4 porcine membranes (13%), and 2 pericardium (6,5%) (Tab. 1). The operating surgeon chooses the type of membrane based on the features of the specific malformation: if a resistant tissue was required, such as in case of AWD, the *fascia lata* was mainly used, if a pliable tissue was required to separate the adjacent sutures, such as in case of EA or SD, the amniotic membrane was preferred.

**Table 1:**
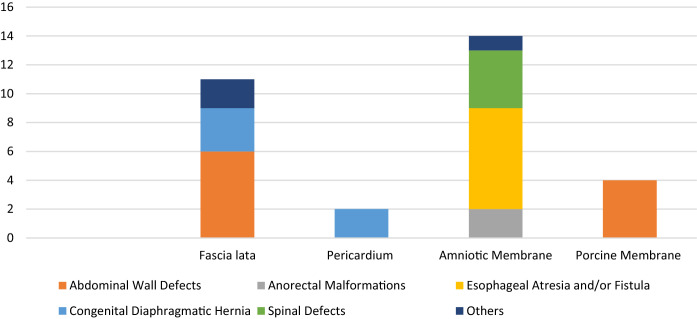
Use of different biological membranes for each condition

### Type of surgical procedure

In AWD patients 6 *fascia lata* and 4 porcine-derived membranes were used in order to close the defects after the reduction of herniated organs into the abdomen. The membranes were fixed to fascial layers with interrupted absorbable suture. In one case, a double patch of *fascia lata* was used both to correct the defect and increase the abdominal wall resistance. One patient, with previous multiple abdominal surgeries for complex ARM (cloaca), was also affected by Prune-Belly Syndrome. At adolescence she required an extensive abdominoplasty and the porcine-derived biological patch was employed. In another patient, affected by CDH, the *fascia lata* was used to allow the closure of the abdominal wall without tension.

In EA/TEF patients the amniotic membrane was used in all cases. The surgical aims were to guarantee a lasting separation between the esophagus and the trachea in case of dehiscence (3 cases) or to cover the esophageal anastomosis under tension during primary repair (4 cases).

In CDH patients the human pericardium was used in 2 cases and *fascia lata* in 3. In 4 patients the membranes were chosen to reinforce the neo-diaphragm and in 1 the *fascia lata* and PTFE patch were used to close the defect.

In ARM patients, the amniotic membrane was used in 2 female patients with cloaca after separation of the urinary and genital tract to consolidate the interface between the neo-vagina and urethra.

Three out of four patients with SD had myelomeningocele that was corrected at birth using the amniotic membrane fixed to the surrounding muscle. In one case, a second amniotic membrane was used to reinforce the first one. The fourth patient was diagnosed with Currarino’s syndrome and underwent the procedure of untethering of the tethered cord. It healed with a postoperative fistula that was repaired with a patch of amniotic membrane.

Finally, the *fascia lata* was used in 2 more patients: 1) after the removal of a bronchogenic cyst in order to reinforce the bronchial wall; 2) in a patient affected by severe inflammatory bowel disease (IBD) and immunological defect with recurrent perineal fistulas. In a patient, with previous neurosurgery for an artero-venous malformation, who presented with persistence of an extradural hematoma, an amniotic membrane was used.

### Post-operative complication

A total of 7 patients (22,5%) presented post-operative complications, and 4 of them required a second procedure. One AWD newborn developed compartment syndrome that required loosening of some stiches, in order to reduce the intra-abdominal pressure, without removal of *fascia lata*. Three EA/FTE patients presented pneumothorax, temporary vocal cord paralysis, and partial dehiscence of anastomosis. One CDH patient had partial wound dehiscence of laparotomy, conservatively treated. Finally, 2 infants with cloaca were reoperated for intestinal occlusion.

### Follow-up

**Fig. 2:**
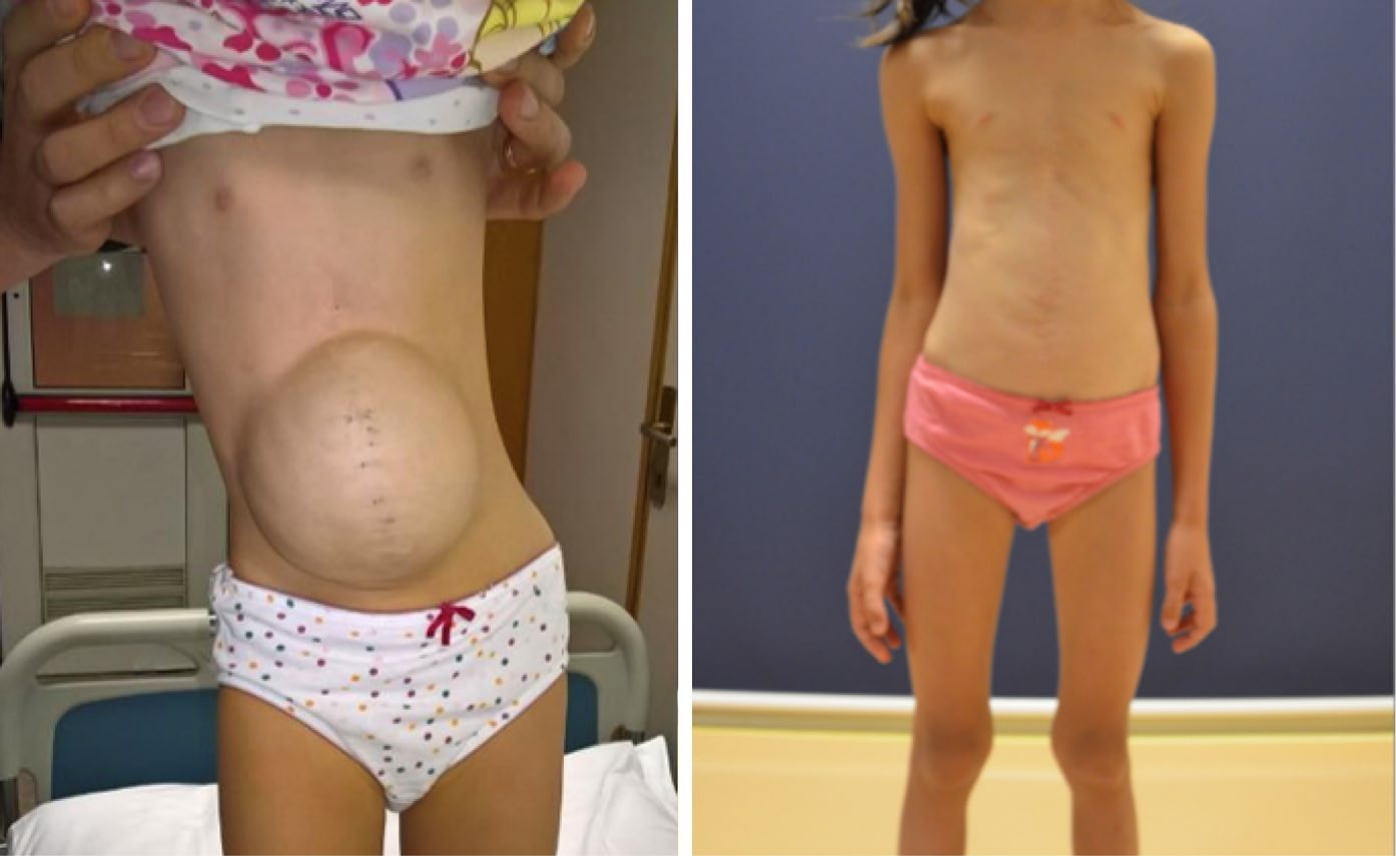
Patient with giant omphalocele before (left) and after (right) correction with a biological patch

**Fig. 3:**
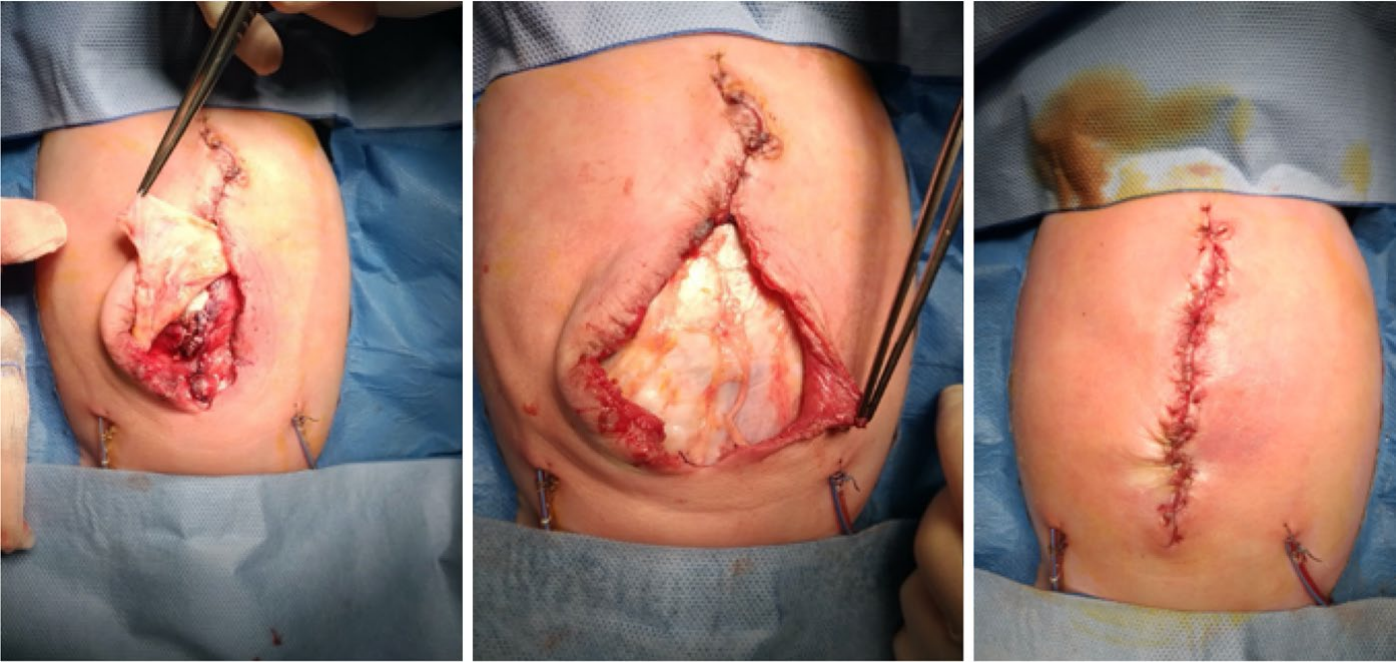
Intraoperative use of a patch of *fascia lata* for the correction of an omphalocele in a newborn

**Fig. 4:**
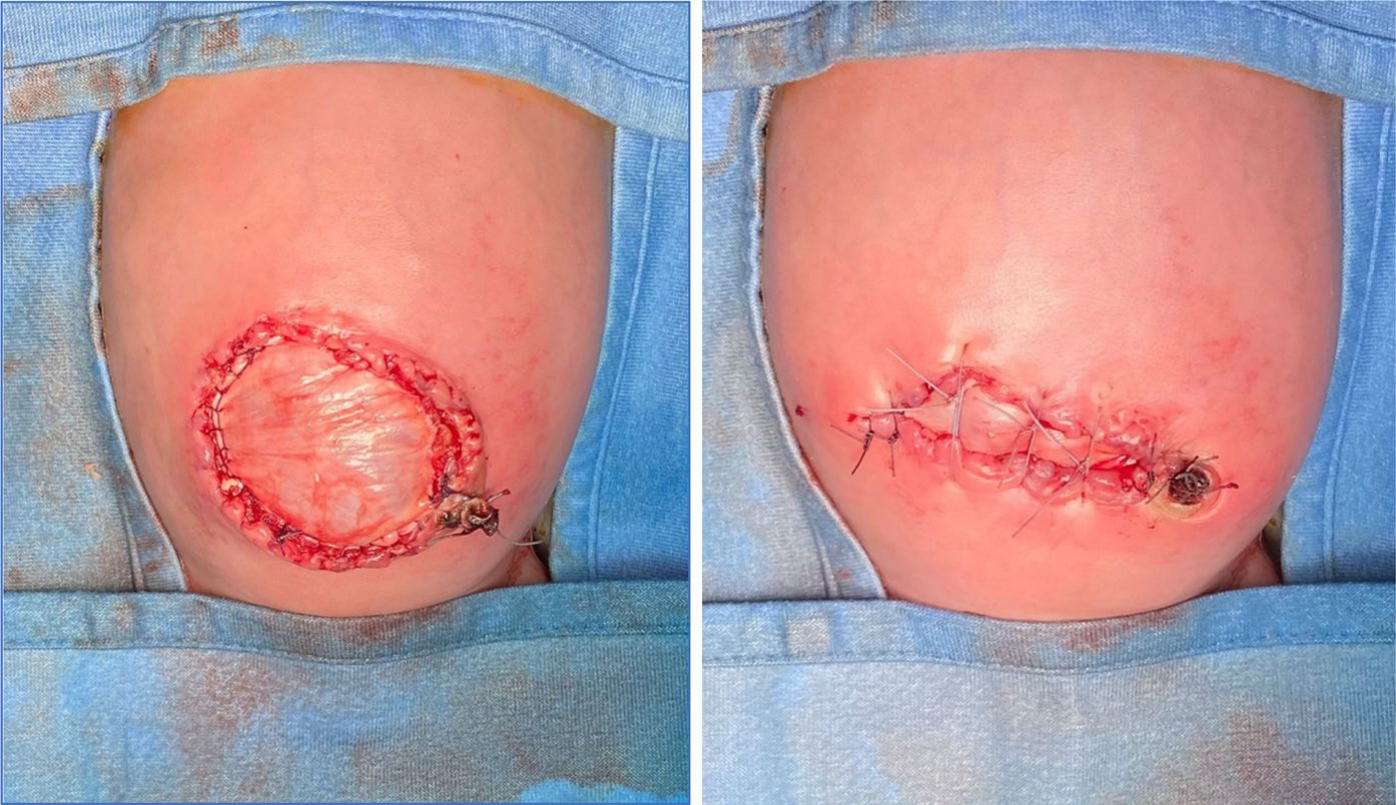
Intraoperative use of a patch of pericardium to correction a AWD (gastroschisis)

**Fig. 5:**
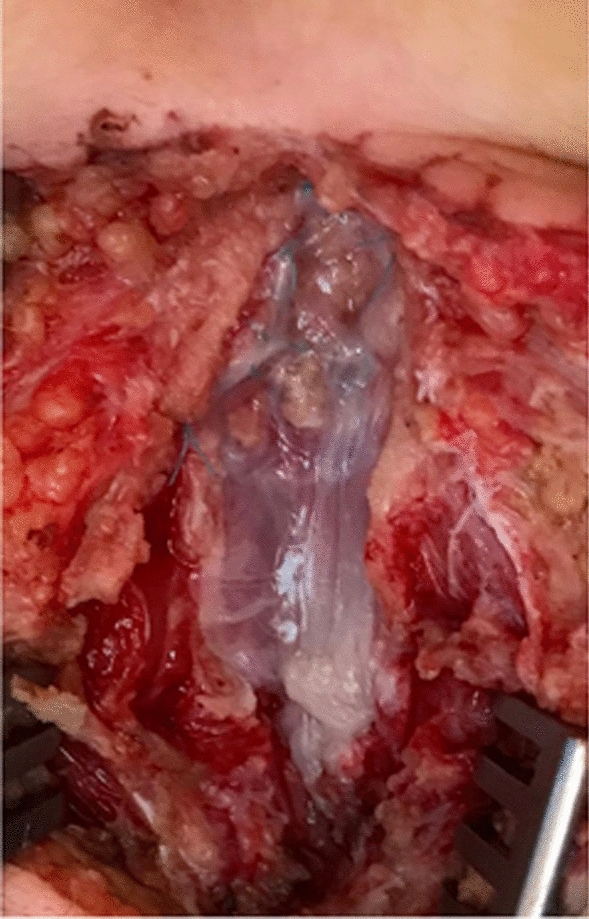
Patient with Currarino Syndrome: interposition of amniotic membrane to repair a persistent postoperative fistula

A post-operative follow-up of minimum 4 months was considered to evaluate both tissue’s performance and host’s tolerance to the biological graft. The mean follow-up was 1021 days (interquartile range: 485,5–1535 days). In one CDH patient, in which the diaphragmatic defect was primarily repaired with *fascia lata*, it was necessary to replace the membrane with a non-absorbable one in order to correct the recurrence. In all the remaining cases, the membranes were not related to any of the complications listed above and functional and aesthetic results were satisfactory. All AWD patients had an excellent aesthetic result at a mean follow-up of 1019,5 days (Fig. 2,3,4). The amniotic membrane was used in all EA patients, both in case or anastomotic dehiscence and separation of adjacent sutures, and it was perfectly tolerated. Similarly, the amniotic membrane used in SD patients to repair the congenital defects and/or fistula recurrence (Fig. 5), and ARM patients to separate two contiguous sutures, did not present any adverse reaction.

## Discussion

The study reports the performance and the tolerance of biological membranes during the surgical repair of major congenital malformations in infants and children. The ideal biological membrane should share mechanical and biological properties identical to the native missing tissue, should be highly resistant to infection after implantation, and should be rapidly integrated in the organ maintaining its original strength and size during remodeling to prevent failure, shrinkage, bulge or stretch (Hammond et al. 2008). The membranes have been used to recreate an anatomical and/or functional separation between organs, to reinforce the anatomical barriers, and to improve the aesthetic results.

The use of biological materials has been widely described in adults. The first reported use of fetal amniotic membrane as a skin substitute was by J.S. Davis in 1910 (Davis 1910). Ever since the use of human amniotic membrane has been validated in a large variety of clinical scenarios including full-thickness burns, chronic ulcers, dural defects, abdominal and urogenital reconstruction, microvascular anastomosis, and esthetic and reconstructive plastic surgery, such as reconstruction of the nasal lining and tympanic membrane (Fairbairn et al. 2014). In spite of the long lasting experience in the adult surgery, the application in pediatric surgery is much more recent (Ahuja et al. 2020). Different synthetic tissues had been previously employed to substitute the missing native tissue in some congenital malformations, such as the Goretex patches for CDH or AWD. However, these materials can be responsible for chronic irritation and infection at the site of implantation (Saxena 2018), have limited durability and plasticity, and do not follow the patient’s growth, thus possibly affecting the function of the substituted tissue. In the pediatric population, the biological materials had been mainly used in case of burns (Puyana et al. 2019). A survey carried out in 2011 demonstrated that the biological membranous dressing performed better than the standard of care on epithelialization rate, length of hospital stay, and pain for the treatment of partial-thickness burns in children, and concluded it is an effective and safe therapy (Vloemans et al. 2014). Another medical field, recently interested by the use of the amniotic membrane, is the pediatric ophthalmology. The anti-inflammatory, anti-microbial, anti-angiogenic and low immunogenicity properties, combined with the relatively easy availability, have favored its use in tissue engineering and development of new therapeutic strategies (Ahmad et al. 2017).

Over the past 10 years, the interest in tissue engineering has increased and different methods to expose the autologous pluripotent cells to the regenerative environments in vitro had been studied. The aim is to promote the tissue expansion in vitro in order to provide autologous grafts to restore the anatomic and/or functional characteristics of a specific organ (Pearson et al. 2017). These tissues reproduce the properties of the derived tissue and may guarantee better mechanical resistance, regenerative potential, and immunological properties. Maintaining host bioreactors, devices that offer sterile conditions and controlled environments similar to those of the human body, these grafts generate tissues very similar to the affected organs thus reducing the inflammatory response after implantation (Saxena 2010). In the future the tissue engineering will represent a valid therapeutic alternative to heterologous biological membranes for the treatment of major congenital malformations.

The human tissues are treated and prepared by authorized organizations, called tissue banks. FBTV is authorized to distribute musculoskeletal tissue, cardiovascular tissue including heart valves, nerves, adipose tissue, and amniotic membrane. FBTV is on duty 24 h and the emergency shipping is guaranteed. This is one of the key factors that allowed to collect the largest reported series of pediatric patients with major congenital malformations in which the biological membranes have been used.

The limits of the study are the heterogeneity of the malformations and the lack of standardization regarding the choice of membranes that is partially based upon the surgeon’s preference. Moreover, the rarity of the malformations contributes to the challenge of standardization toward indications, type of membranes, and proposals of randomized trials.

In our experience, the human and the porcine membranes demonstrated to be well tolerated also at a medium-term follow up as well as their function resulted in a satisfying performance. Moreover, the esthetical results turned out to be excellent.

The variety of membranes the tissue banks can prepare allow to choose the most suitable one for each malformation. Our data confirmed the amniotic membrane should be used when the damaged tissue needs to be covered after the repair and/or separated from other tissues; the *fascia lata* and the pericardium are excellent and robust supports that should be used to reinforce the sutures under tension or to fill the lack of tissue, such as in case of large AWD. Caution is needed in case of large CDH defects for which no enough evidence exists in favor of absorbable materials (Gasior and St Peter 2012).

## Conclusion

The treatment of congenital malformations is still challenging in pediatric surgery because of the lack of original tissue and the need for a multidisciplinary treatment. The use of human or porcine membranes in this case series has produced promising results, even if they have been mostly used in case of complications of the primary repair or as reinforcement of the suture. This preliminary experience is very encouraging and can set the base for future investigations.
